# The Association Between Internet Use and Co-occurring Health Care Needs: Cross-Sectional Study in China

**DOI:** 10.2196/67484

**Published:** 2025-04-17

**Authors:** Yiqing Xing, Xuejiao Liu, Liang Zhang, Ruibo He

**Affiliations:** 1 School of Political Science and Public Administration Wuhan University Wuhan China; 2 School of Medicine and Health Management Tongji Medical College Huazhong University of Science and Technology Wuhan China; 3 School of Finance and Public Administration Hubei University of Economics Wuhan China

**Keywords:** internet use, co-occurring health care needs, physical exercise, health status, Chinese residents

## Abstract

**Background:**

The need for health care underpins health care service provision and serves as the foundation for enhancing service capacity and allocating resources. Health care needs are influenced by health, social, and economic conditions and may exhibit different characteristics over time. However, previous studies have primarily focused on specific populations or types of needs, overlooking the diversity and complexity of residents’ health care requirements. Furthermore, as informatization becomes a defining aspect of modern social development, the impact of internet utilization on the co-occurrence of health care needs remains unclear.

**Objective:**

This study aims to determine the co-occurrence of health care needs among residents in China, explore the relationship between internet use frequency and co-occurring health care needs, and analyze the potential pathways of influence.

**Methods:**

Data were obtained from the “Survey on Chinese Residents’ Health Services Needs in the New Era,” conducted from July to August 2018, yielding a sample of 12,513 individuals. An association rule learning algorithm was used to analyze the characteristics of co-occurring health care needs among Chinese residents, while a generalized linear model was used to examine the relationship between internet use frequency and co-occurring health care needs. Additionally, physical exercise and health status were selected as mediating variables, and their mediating effects were assessed using a path analysis model.

**Results:**

A substantial proportion of the surveyed population (8601/12,513, 68.74%) had 2 or more co-occurring health care needs, with a lower percentage among rural residents (4045/6053, 66.83%) compared with urban residents (4556/6460, 70.53%). Frequent internet users tended to have more co-occurring health care needs (β=.895, SE 0.019, *P*<.001). The results indicated a positive relationship between internet use frequency and both improved physical exercise (β=.121, *P*<.001) and health status (β=.026, *P*<.001). Notably, a high level of physical exercise was associated with an increase in co-occurring health care needs (β=.087, *P*<.001). By contrast, a significant negative correlation was observed between health status and co-occurring health care needs (β=-.787, *P*<.001), indicating that these needs decreased as health status improved.

**Conclusions:**

The findings highlight the need for health policy makers and health care providers to address evolving health care needs and the impact of information technology on these needs. Furthermore, health care providers must adapt their services and delivery methods to meet residents’ co-occurring health care needs. Meanwhile, policy makers and service managers should ensure that service delivery keeps pace with residents’ changing needs through resource allocation, health insurance payment reforms, and performance incentives.

## Introduction

### Chinese Residents’ Changing Health Care Needs and Imbalance in Supply and Demand

Meeting the health care needs of a population forms the foundation for a country or a region to promote the effective use of medical resources and ensure a reasonable supply of services [1], as well as to optimize population health levels and enhance social welfare [2]. With the development of the social economy and improvements in living standards, residents’ health care needs have undergone structural changes, leading to greater diversity and complexity in co-occurring health care needs [3,4]. In this research, the concept of co-occurring needs is defined as “multiple health care needs that exist simultaneously in an individual” [5], such as myopia treatment, healthy lifestyle guidance, and psychological disorder relief for adolescents, as well as chronic disease management, traditional Chinese medicine, and home-based care for older adults. However, the current level of service provision fails to match the diversity of needs and tends to focus on individual aspects, particularly medical services in large hospitals. Residents’ co-occurring needs, such as health guidance and disease prevention, remain unmet, and corresponding service providers are lagging in the development of such services. One study concluded that existing service supply systems may lack the capacity to meet residents’ diverse health care requirements in this new era [6]. The imbalance between service supply and demand has become a widespread issue, largely due to a limited understanding and appreciation of residents’ health care service needs. Additionally, health care providers and policy makers, who serve as health resource users and distributors, respectively, contribute to this imbalance. Despite substantial resource investments, these have yet to be effectively translated into the services residents need, leading to the wastage of valuable health resources and reducing the accessibility of other essential services [7]. Therefore, in the context of the “Healthy China Strategy,” significant changes in residents’ health care needs must be addressed. In particular, research should focus on investigating the co-occurrence structure of health care needs, as well as the current situation and influencing factors in this new era. The findings could serve as a valuable reference for developing health care service package policies in China, helping to address residents’ health care needs and achieve a balance between health care service supply and demand.

### Internet Use and Health Care Needs

While there are multiple ways to address the imbalance between health care supply and demand, the rapid penetration of internet technology offers new possibilities [8,9]. According to the 51st Statistical Report on China’s Internet Development [10], released by the China Internet Network Information Center, the number of Chinese internet users reached 1.067 billion in December 2022, with an internet penetration rate of 75.6%, showing a year-on-year growth trend. With the rapid advancement of information technology, the internet has become a key channel for residents to access knowledge. According to a report by the China Internet Network Information Center, the number of Chinese online medical service users surged to 298 million, reflecting a year-on-year increase of 38.7%. As of December 2021, this growth rate made online medical services the fastest-growing service category in terms of usage [11]. The growing popularity of the internet may have increased residents’ health care needs while also enabling them to meet these needs through online medical consultations, telemedicine, and hospital websites [12].

Previous studies have shown that the internet has the potential to expand individual needs and drive the evolution of need structures [13], including those of older adults, maternal, and adolescent populations. One study found that older adults use the internet to access health information from various platforms, primarily due to the convenience and cost-effectiveness of these resources. This trend has contributed to the growing demand for health care services such as preventive care, traditional Chinese medicine, and lifestyle guidance [14]. Moreover, internet usage has been linked to an increased demand for outpatient services and a higher volume of doctor visits among older adults [15,16].

Studies on the health care needs of maternal residents have reported that internet applications play a vital role in meeting this population’s health care needs [17]. Additionally, the powerful information retrieval capabilities of internet technology have increased the demand for online access to maternal health care services among 89% of pregnant women [18]. For individuals with chronic diseases, such as diabetes, internet usage has been found to reduce the need for outpatient services [19]. Other factors, such as demographic characteristics, socioeconomic status, and service accessibility, have also been found to influence health care needs across different populations [20,21]. However, current research on health care needs typically focuses on the individual needs of specific populations, largely overlooking the co-occurrence of multiple health care needs. In addition, the extent to which internet use affects co-occurring health care needs remains unclear.

Furthermore, the mechanism underlying the association between internet use and co-occurring health care needs has yet to be explored in depth. Potential pathways linking internet use to co-occurring health care needs may include physical exercise and health status. On the one hand, internet use promotes both physical activity frequency and overall health. The diffusion of internet-based information technology has shaped health concepts and influenced residents’ behaviors by facilitating information dissemination [22,23], thereby encouraging physical exercise [24]. Meanwhile, the relationship between internet use and health levels has been extensively examined. The internet provides easy access to health-related knowledge and information [25], enabling residents to optimize their health input mix, improve their physical health in a targeted manner, and enhance their health self-management abilities [26,27]. Additionally, internet technology allows individuals in need of medical services to achieve better treatment outcomes and improved health outputs [28,29]. However, some studies have argued that excessive internet use can lead to the development of unhealthy behaviors and ultimately negatively impact an individual’s health [30,31]. On the other hand, physical exercise and health status have been shown to be important factors influencing residents’ health care needs. Research suggests that individuals who engage in physical exercise more frequently tend to have higher health literacy and a greater need for traditional Chinese medicine specialty health management services [32,33]. Compared with residents who do not engage in physical exercise, those who exercise regularly may have a higher demand for outpatient and mental health services [34,35]. Regarding the relationship between health status and health care needs, studies have found that lower health levels are associated with a greater need for mental health services, whereas better health status corresponds to a lower demand for health care services [36]. Therefore, based on relevant studies, this paper selects physical exercise and health status as mediating variables to explore the correlation mechanism between internet use frequency and co-occurring health care needs.

### Research Objectives and Hypotheses

To address gaps in the literature, we used survey data from 4 counties/districts in China to examine residents’ co-occurring health care needs in the context of the information age. Specifically, our study aims to explore the relationship between internet use frequency and co-occurring health care needs among Chinese residents, as well as whether physical exercise and health status moderate this relationship. We hypothesize that more frequent internet use is associated with a higher incidence of co-occurring health care needs among residents. Furthermore, we hypothesize that frequent internet use increases health care needs by promoting physical exercise, while it decreases health care needs by improving health status.

## Methods

### Study Design and Sample Selection

We obtained the data for this study from the “Survey on Chinese Residents’ Health Service Needs in the New Era,” conducted by the Chinese Rural Health Services Research Centre from July to August 2018 [37]. The survey took into account the socioeconomic and cultural factors, customs, and geographic environments of 2 urban districts (Futian District in Eastern China and Xiling District in Central China) and 2 rural counties (Danyang County in Central China and Sinan County in Western China) [38].

We used multistage stratified random sampling to obtain a representative sample from each district and county, collecting data at 4 levels: district (county), street (township), community (village), and household. First, we selected 5 streets from each district that were in close proximity to the district medical center. Next, we randomly selected 5 townships from each county that were near the county hospital, resulting in a total of 10 streets and 10 townships. Second, we randomly selected 6 communities and villages from each street and township based on their proximity to the village clinic, resulting in a total of 30 communities and 30 villages. Third, by referring to the Fifth National Health Services Survey of China (2013) [39], we determined a minimum sample size of 3583 residents for each surveyed area. This was calculated using a chronic disease prevalence of 21.338% in the overall population, a design effect of 2.5%, and a confidence level of 95%. In China, a household typically consists of around 3 members [40]; therefore, we planned to survey 1200 households in each community and village. To account for potential refusals or invalid responses, we conducted resampling to survey additional households, ensuring that the final sample size met the estimated requirements. All members of the sampled households participated in face-to-face interviews and a questionnaire survey.

The survey strictly controlled the on-site research process, requiring us to confirm that the responding household members included the head of the family and at least half of the adult household members before starting the survey. If these criteria were not met, we scheduled another appointment or selected an alternative household. At the operational level, the survey achieved a self-response rate of over 70% (11,198/15,126, 74.03%), indicating a reliable representation of individuals and their health care needs. We closely monitored the self-response rate of the questionnaire to ensure accuracy. The survey data were double-entered using EpiData 3.1 (EpiData Association) and underwent multiple rounds of cleaning. After further screening based on the study design, we obtained a final sample of 5547 households and 15,126 respondents. For our analysis, we included individuals aged 15 years or older. After eliminating invalid cases and those with missing responses, we arrived at a final sample of 12,513 valid respondents.

### Ethical Considerations

The questionnaire design and study protocol were approved by the Ethics Committee of Tongji Medical College, Huazhong University of Science and Technology (approval number IORG0003571). The research content and procedures complied with national and state biomedical ethical requirements. All participants provided written informed consent after receiving information to ensure a full understanding of the study’s purpose and potential outcomes. Participation was voluntary and uncompensated, and all study data were kept confidential. To mitigate potential biases in self-report surveys, the study implemented several strategies, including ensuring respondent anonymity and emphasizing the importance of honest self-reporting.

### Variables

#### Outcome Variable

In this study, the outcome variable was co-occurring health care needs. We recorded all residents’ self-reported health care service needs, covering a wide range of categories—from routine medical needs (eg, outpatient services) to population-specific needs (eg, older adult, maternal, and pediatric care)—to ensure comprehensive coverage of different groups. We measured residents’ health care needs using selected questions from the questionnaire: “Do you need outpatient services within two weeks?” “Do you need traditional Chinese medicine?” “Do you need inpatient services?” “Do you need health guidance?” “Do you need physical exercise guidance?” “Do you need mental health services?” “Do you need network health information services?” “Do you need older adult health management services?” “Do you need chronic disease management services?” “Do you need rehabilitation services?” “Do you need maternal health management services?” and “Do you need child health management services?” Each of the 12 questions had a binary response: “yes” (assigned a value of 1) or “no” (assigned a value of 0). The total number of co-occurring health care needs was calculated by summing these values, where a higher total indicated greater co-occurring health care needs among Chinese residents.

#### Explanatory Variable

In this research, the explanatory variable was internet use frequency. To measure this, we asked participants the question: “How often do you use the internet to obtain health information (including via computer or smartphone)?” Participants selected 1 of 3 responses: “never,” “occasionally,” or “frequently.” We then categorized these responses into 3 groups, assigning values of 1 and 3 to indicate low and high internet use frequency, respectively.

#### Mediating Variables

In this study, the mediating variables were physical exercise and health status. To measure physical exercise, we asked participants: “How many times do you exercise in a week?” We then categorized physical exercise into 3 groups based on the average number of times respondents exercised per week. Following the literature [41], individuals who exercised 2 times a week or fewer, 3-6 times a week, and 7 times or more per week were classified into low-, medium-, and high-level groups, respectively. Second, we assessed health status based on individuals’ self-evaluation of mobility, self-care, usual activities, pain/discomfort, and anxiety/depression. We used the EuroQol Five Dimensions Questionnaire index scores and applied the time trade-off method to quantify respondents’ health status.

#### Control Variables

Following the literature [20,23], we included additional factors that may influence residents’ co-occurring health care needs, such as demographic and socioeconomic attributes. The demographic characteristics considered as covariates included gender, age, marital status, educational level, and household registration. For socioeconomic characteristics, we analyzed factors that may influence residents’ co-occurring health care needs, including employment status, income level, family type, health insurance coverage, and access to services. Income levels were categorized into 5 groups based on respondents’ average annual incomes: low (<RMB 20,000; RMB 1=US $0.14), lower middle (RMB 20,000-50,000), middle (RMB 50,000-100,000), upper middle (RMB 100,000-150,000), and high (>RMB 150,000). The details of these variables are presented in Table 1.

**Table 1:** Coding of variables.

Variable name			Variable definition		Attribute
**Outcome variable**					
	Co-occurring health care needs	Number of health services required by an individual at the same time.		Numerical values, ranging from 1 to 11	
**Explanatory variable**					
	Frequency of internet use	The frequency of individuals accessing health information through the internet, including computers and smartphones.		1=rarely used, 2=occasionally used, and 3=frequently used	
**Mediating variables**					
	Physical exercise	The calculation was based on the average frequency of physical exercise per week in the last 30 days.		1=low level, 2=moderate level, and 3=high level	
	Health status	The EQ-5D time trade-off value obtained by comprehensive calculation.		1=low level, 2=moderate level, and 3=high level	
**Control variables**					
	Gender	Participant’s gender.		0=female and 1=male	
	Age	Participant’s age (range over 15 years).		1=15-44 years, 2=45-64 years, and 3=65 years and above	
	Marital status	The spouse status of the participant.		0=without spouse and 1= with spouse	
	Education level	The education level of the participant.		1=uneducated, 2=primary education, 3=secondary education, 4=higher education, and 5=postgraduate and above	
	Household registration	The household registration type of the participant.		0=rural and 1=urban	
	Employment status	Employment status of the participant.		1=unemployed, 2=schooled, 3=retired, and 4=employed	
	Income level	Average annual income of individuals.		1=low income, 2=lower middle income, 3=middle income, 4=upper middle income, and 5=high income	
	Family type	Whether the participant’s family was certified poor by the government.		0=no and 1=yes	
	Health insurance	Whether the participant had participated in social health insurance or commercial health insurance.		0=no and 1=yes	
	Service accessibility	The duration to the nearest medical facility.		1=within 15 minutes and 2=over 15 minutes	

### Statistical Analysis

We conducted data analysis using Stata 15 (StataCorp) and SPSS version 25 (IBM Corp.). First, we applied the association rule learning algorithm to examine residents’ co-occurring health care needs. When calculating co-occurring health care need items, we focused on combinations of needs that appeared in over 100 instances of internet use. Second, we performed a descriptive analysis to summarize the sample characteristics and compared differences between urban and rural groups. Third, we applied a generalized linear model to empirically analyze the relationship between internet use frequency and co-occurring health care needs. We reported regression coefficients, SEs, *P* values, 95% CIs, and McFadden *R*^2^. Additionally, we assessed multicollinearity using variance inflation factors and evaluated normality through a residual histogram. Finally, we conducted a path analysis to examine the mediating effects of physical exercise and health status on the relationship between internet use frequency and co-occurring health care needs.

## Results

### Current State of Co-Occurring Health Care Needs

Among the general population, multiple co-occurring health care needs emerged from a combination of 8 general health care services. Of those with co-occurring health care needs (N=4401), 2189 (49.74%) had 2 needs, 1611 (36.61%) had 3 needs, and 510 (11.59%) had 4 needs. Only 91 (2.07%) had 5 or more co-occurring health care needs. Furthermore, after identifying critical groups—such as older adults, individuals requiring maternal postnatal health management, and those needing chronic disease management—we found that 3122 out of 9339 (33.43% of the total sample) individuals required 2 concurrent health interventions. Additionally, 2880 out of 9339 (30.84%) individuals required 3 types of concurrent health care services, 1767 out of 9339 (18.92%) individuals required 4 types, and 1580 out of 9339 (16.92%) individuals required 5 or more concurrent health care services.

The most common combination of health care services among respondents was “physical exercise guidance + network health information service + traditional Chinese medicine” (n=859), followed by “physical exercise guidance + network health information service” (n=620). The third most common combination was “network health information service + traditional Chinese medicine” (n=469). Other relatively common health care service combinations were “health guidance + physical exercise guidance + network health information service + traditional Chinese medicine” (n=356), “physical exercise guidance + traditional Chinese medicine” (n=285), “health guidance + physical exercise guidance” (n=281), and “health guidance + physical exercise guidance + network health information service” (n=204).

### Descriptive Analysis of Key Variables and Sample Characteristics

Table 2 presents the characteristics of the respondents. Among the 12,513 participants, 8601 (68.74%) had more than 2 concurrent health care needs. Rural residents had a lower percentage of co-occurring health care needs (4045/6053, 66.83%) compared with urban residents (4556/6460, 70.53%). Additionally, more than half of the participants (7186/12,513, 57.43%) did not use the internet, while 3461 (27.66%) used it occasionally, and 1866 (14.91%) used it frequently. Moreover, a significantly higher proportion of rural residents did not use the internet (4840/6053, 79.96%; *P*<.001) compared with urban residents (2346/6460, 36.32%). Similarly, the proportion of high-frequency internet users was much lower among rural residents (391/6053, 6.46%) than among urban residents (1475/6460, 22.83%).

The overall physical exercise levels in the sample population were normal, with urban residents engaging in significantly more exercise than rural residents (*P*<.001). Among rural residents, the proportion of those who rarely exercised (4301/6053, 71.06%) was higher than among urban residents (1373/6460, 21.25%), whereas the proportion of those who exercised frequently (984/6053, 16.26%) was significantly lower than among urban residents (3138/6460, 48.58%; *P*<.001). Regarding health status, 10,414 of 12,513 (83.23%) respondents were in good health, while only 94 of 12,513 (0.75%) reported poor health. A comparison between the 2 groups revealed statistically significant differences in co-occurring health care needs (*P*<.001), internet use frequency (*P*<.001), physical exercise (*P*<.001), and health status (*P*<.001).

Table 2 presents the respondents’ demographic and socioeconomic characteristics. Among them, 6432 of 12,513 (51.40%) were female, and 10,142 of 12,513 (81.05%) were married. The majority belonged to the 45-64-year age group (4966/12,513, 39.69%) or the 15-44-year age group (4394/12,513, 35.12%). Nearly half had completed secondary education (5879/12,513, 46.98%), while only 211 of 12,513 (1.69%) had a postgraduate degree or higher. In terms of socioeconomic characteristics, the majority of respondents were employed at the time of the survey (7750/12,513, 61.94%). Regarding income, 2831 of 12,513 (22.62%) belonged to the middle-income group, 2474 of 12,513 (19.77%) to the low-income group, and 2232 of 12,513 (17.84%) to the upper-middle-income group. Most respondents came from poor households (11,347/12,482, 90.91%) and had health insurance (12,365/12,478, 99.09%). Additionally, most had access to a health care provider within 15 minutes or less (11,177/12,513, 89.32%).

In summary, a comparative analysis of the 2 groups revealed statistically significant differences in age (*P*<.001), marital status (*P*<.001), household registration type (*P*<.001), employment status (*P*<.001), income level (*P*<.001), and access to services (*P*<.001). However, no statistically significant differences were found in gender (*P*=.63) or family type (*P*=.21).

**Table 2:** Description of sample characteristics.

Variables		Overall (N=12,513)	Rural (n=6053)	Urban (n=6460)	*P* value
**Co-occurring health care needs, n (%)**					<.001
	No	3912 (31.26)	2008 (33.17)	1904 (29.47)	
	Yes	8601 (68.74)	4045 (66.83)	4556 (70.53)	
**Frequency of internet use, n (%)**					<.001
	Rarely used	7186 (57.43)	4840 (79.96)	2346 (36.32)	
	Occasionally used	3461 (27.66)	822 (13.58)	2639 (40.85)	
	Frequently used	1866 (14.91)	391 (6.46)	1475 (22.83)	
**Physical exercise, n (%)**					<.001
	Low level	5673(45.34)	4301 (71.06)	1372 (21.24)	
	Moderate level	2718 (21.72)	768 (12.69)	1950 (30.19)	
	High level	4122 (32.94)	984 (16.26)	3138 (48.58)	
**Health status, n (%)**					<.001
	Low level	94 (0.75)	71 (1.17)	23 (0.36)	
	Moderate level	1917 (15.32)	1586 (26.20)	331 (5.12)	
	High level	10,414 (83.23)	4372 (72.23)	6042 (93.53)	
**Gender, n (%)**					.63
	Female	6081 (48.60)	2970 (49.07)	3111 (48.16)	
	Male	6432 (51.40)	3083 (50.93)	3349 (51.84)	
**Age (years), n (%)**					<.001
	15-44	4394 (35.12)	1523 (25.16)	2871 (44.44)	
	45-64	4966 (39.69)	2846 (47.02)	2120 (32.82)	
	65 and above	3153 (25.20)	1684 (27.82)	1469 (22.74)	
**Marital status, n (%)**					.21
	Without spouse	2371 (18.95)	1170 (19.33)	1201 (18.59)	
	With spouse	10,142 (81.05)	4883 (80.67)	5259 (81.41)	
**Education level, n (%)**					<.001
	Uneducated	1282 (10.25)	1068 (17.64)	214 (3.31)	
	Primary education	2650 (21.18)	2095 (34.61)	555 (8.59)	
	Secondary education	5879 (46.98)	2707 (44.72)	3172 (49.10)	
	Higher education	2491 (19.91)	176 (2.91)	2315 (35.84)	
	Postgraduate and above	211 (1.69)	7 (0.12)	204 (3.16)	
**Employment status, n (%)**					<.001
	Unemployed	1606 (12.83)	1048 (17.31)	558 (8.64)	
	Schooled	567 (4.53)	303 (5.01)	264 (4.09)	
	Retired	2590 (20.70)	364 (6.01)	2226 (34.46)	
	Employed	7750 (61.94)	4338 (71.67)	3412 (52.82)	
**Income level^a^, n (%)**					<.001
	Low income	2596 (20.75)	2433 (40.19)	163 (2.52)	
	Lower middle income	2427 (19.40)	2009 (33.19)	418 (6.47)	
	Middle income	2831 (22.62)	1183 (19.54)	1648 (25.51)	
	Upper middle income	2232 (17.84)	337 (5.57)	1895 (29.33)	
	High income	2427 (19.40)	91 (1.50)	2336 (36.16)	
**Family type, n (%)**					<.001
	Poor family	11,347 (90.68)	4979 (82.26)	6368 (98.58)	
	Nonpoor family	1135 (9.07)	1061 (17.53)	74 (1.15)	
**Health insurance, n (%)**					<.001
	No	113 (0.90)	29 (0.48)	84 (1.30)	
	Yes	12,365 (98.82)	6004 (99.19)	6361 (98.47)	
**Service accessibility, n (%)**					<.001
	Within 15 minutes	11,117 (89.84)	4834 (79.86)	6283 (97.26)	
	Over 15 minutes	1297 (10.37)	1172 (19.36)	125 (1.93)	

^a^Income levels were categorized into 5 groups based on respondents’ average annual incomes: low (<RMB 20,000; RMB 1=US $0.14), lower middle (RMB 20,000-50,000), middle (RMB 50,000-100,000), upper middle (RMB 100,000-150,000), and high (>RMB 150,000).

### Association Between Internet Use Frequency and Co-Occurring Health Care Needs

Table 3 presents the regression results of factors that may influence co-occurring health care needs. A positive correlation was observed between internet use frequency and co-occurring health care needs (β=.895, SE 0.019, *P*<.001, 95% CI 0.857-0.934). As respondents’ internet use frequency increased, their co-occurring health care needs also increased proportionally. Regarding demographic attributes, being male (β=.185, SE 0.038, *P*=.001, 95% CI 0.110-0.261), older (β=.296, SE 0.046, *P*<.001, 95% CI 0.206-0.386), and married (β=.106, SE 0.030, *P*=.001, 95% CI 0.046-0.167) were associated with higher co-occurring health care needs. By contrast, household registration had a negative impact on co-occurring health care needs (β=–.440, SE 0.036, *P*=.002, 95% CI 0.512-0.369).

Regarding socioeconomic characteristics, employment status significantly increased co-occurring health care needs among residents (β=.290, SE 0.072, *P*=.001, 95% CI 0.148-0.433), indicating that employed residents had more co-occurring health care needs than unemployed residents. Additionally, high-income (β=.121, SE 0.048, *P*=.01, 95% CI 0.028-0.215) and upper-middle-income (β=.365, SE 0.051, *P*<.001, 95% CI 0.266-0.464) individuals had more co-occurring health care needs than lower-income individuals. Furthermore, those from poor families (β=.746, SE 0.128, *P*<.001, 95% CI 0.494-0.997) had more co-occurring health care needs than those from rich families. Besides, having health insurance (β=.249, SE 0.125, *P*=.05, 95% CI 0.003-0.495) and considerable access to medical services (β=.151, SE 0.178, *P*<.001, 95% CI 0.114-0.187) were positively associated with a higher number of co-occurring health care needs among residents.

In addition, a series of diagnostic tests were conducted to assess the generalized linear model. As shown in Table 3, McFadden *R*^2^ was 0.368, indicating a good model fit. The results of the multicollinearity test, presented in Multimedia Appendix 1, show that all variance inflation factors were below 10, suggesting that multicollinearity is not a concern. Furthermore, the residual histogram in Multimedia Appendix 2 supports the normality assumption, as the residuals exhibit an approximately normal distribution.

**Table 3:** Regression results of the effect of internet use frequency and other covariates on co-occurring health care needs.

Variables		*β*	SE	*P* value	95% CI
Frequency of internet use		.895	0.019	<.001	0.857 to 0.934
Gender		.185	0.038	.001	0.110 to 0.261
Age		.296	0.046	<.001	0.206 to 0.386
Marital status		.106	0.030	.001	0.046 to 0.167
**Education level^a^**					
	Primary education	.145	0.087	.09	–0.026 to 0.317
	Secondary education	.020	0.092	.83	–0.201 to 0.161
	Higher education	.071	0.114	.53	–0.152 to 0.294
	Postgraduate and above	.166	0.207	.42	–0.240 to 0.572
Household registration		–.440	0.036	.002	–0.512 to –0.369
**Employment status^b^**					
	Schooled	.069	0.079	.39	–0.086 to 0.223
	Retired	.172	0.114	.13	–0.397 to 0.052
	Employed	.290	0.072	<.001	0.148 to 0.433
**Income level^c,d^**					
	Lower middle income	.034	0.038	.38	–0.041 to 0.109
	Middle income	.070	0.041	.09	–0.010 to 0.150
	Upper middle income	.121	0.048	.011	0.028 to 0.215
	High income	.365	0.051	<.001	0.266 to 0.464
Family type		.746	0.128	<.001	0.494 to 0.997
Health insurance		.249	0.125	.05	0.003 to 0.495
Service accessibility		.151	0.178	<.001	0.114 to 0.187

^a^The reference group used for the education level was uneducated individuals.

^b^The reference group used for the employment status was individuals who were unemployed.

^c^The reference group for the income level was individuals who were classified as low income.

^d^Income levels were categorized into 5 groups based on respondents’ average annual incomes: low (<RMB 20,000; RMB 1=US $0.14), lower middle (RMB 20,000-50,000), middle (RMB 50,000-100,000), upper middle (RMB 100,000-150,000), and high (>RMB 150,000).

### Path Analysis Between Internet Use Frequency and Co-Occurring Health Care Needs

Table 4 presents the influence path of the association between internet use frequency and co-occurring health care needs. First, gender (β=.087, SE 0.011, *P*<.001), marital status (β=.060, SE 0.014, *P*<.001), educational level (β=.214, SE 0.008, *P*<.001), household registration (β=.257, SE 0.016, *P*<.001), and income level (β=.046, SE 0.087, *P*<.001) were significantly positively correlated with internet use frequency. Residents who were male, married, highly educated, had an urban household registration, and had high income levels used the internet frequently. However, age was negatively correlated with internet use frequency (β=–.192, SE 0.008, *P*<.001), indicating that older residents used the internet less frequently.

**Figure 1:**
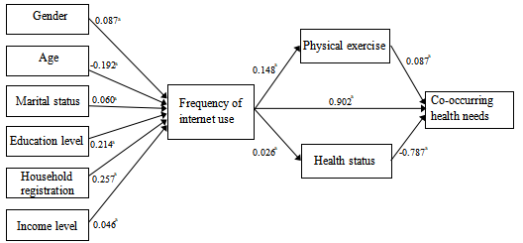
Path diagram of the frequency of internet use affects co-occurring health needs. ^a^P<.001.

Second, regarding the effect of internet use frequency on physical exercise and health status, a significant positive correlation was found between internet use frequency and physical exercise (β=.121, SE 0.011, *P*<.001). This suggests that as residents’ internet use frequency increased, their weekly exercise frequency also increased, indicating an improvement in physical activity. Similarly, internet use frequency was positively correlated with health status (β=.026, SE 0.049, *P*<.001), suggesting that higher internet use was associated with better health among residents. Furthermore, physical exercise had a positive effect on co-occurring health care needs (β=.087, SE 0.054, *P*<.001), indicating that higher levels of physical exercise were associated with greater co-occurring health care needs. However, a significant negative correlation was found between health status and co-occurring health care needs (β=–.787, SE –0.213, *P*<.001), suggesting that better health status was linked to fewer co-occurring health care needs (Figure 1).

**Table 4:** Regression coefficients of path analysis.

Explanatory variable → outcome variable	β1^a^	β2^b^	SE	*P* value
Gender → frequency of internet use	.087	.059	0.011	<.001
Age → frequency of internet use	–.192	–.200	0.008	<.001
Marital status → frequency of internet use	.060	.033	0.014	<.001
Education level → frequency of internet use	.214	.268	0.008	<.001
Household registration → frequency of internet use	.257	.174	0.016	<.001
Income level → frequency of internet use	.046	.006	0.087	<.001
Frequency of internet use → physical exercise	.148	.121	0.011	<.001
Frequency of internet use → health status	.026	.049	0.005	<.001
Frequency of internet use → co-occurring health care needs	.902	.457	0.019	<.001
Physical exercise → co-occurring health care needs	.087	.054	0.015	<.001
Health status → co-occurring health care needs	–.787	–.213	0.034	<.001

^a^The results of unstandardized coefficients.

^b^The results of standardized coefficients.

## Discussion

### Principal Findings

Our study offers a novel perspective by examining the prevalence of multiple co-occurring health care needs among Chinese residents, thereby expanding the existing literature, which primarily focuses on singular health care needs. This study explores the current status of co-occurring health care needs among Chinese residents and investigates the relationships and pathways between residents’ internet use frequency and co-occurring health care needs, using data from the “Survey on Chinese Residents’ Health Service Needs in the New Era” conducted across 4 districts. The results revealed that co-occurring health care needs were common among Chinese residents, with 8601 of 12,513 (68.74%) participants having more than 2 concurrent needs. Key populations, including individuals with chronic diseases, pregnant women, and children, exhibited more diverse health care needs compared with the general population. Among specific health care services, preventive care was the most commonly required. The most frequent combination of services included physical exercise guidance, network health information, and traditional Chinese medicine. These findings can serve as a reminder to policy makers to align system planning, resource allocation, and service management with the evolving characteristics of residents’ health care needs. By doing so, they can create a more responsive service environment and guide service development effectively. Additionally, the findings can help service providers adapt their service content and delivery modes in this digital age, ensuring a better balance between supply and demand in the health care system.

We also observed a notable difference in co-occurring health care needs between urban and rural residents, with the latter having significantly fewer co-occurring needs than the former. This finding aligns with previous research [42]. In China, the persistent urban-rural gap has resulted in better health care access and higher levels of physical exercise among urban residents compared with their rural counterparts [43]. Urban residents in our study demonstrated a considerable need for preventive care services, such as network health information, mental health support, and physical exercise guidance. Providing these services is essential for disease prevention and overall well-being in urban populations. By contrast, rural residents had a greater demand for disease diagnosis and curative services, including but not limited to chronic disease management, outpatient care, and inpatient treatment. Rural residents may perceive that the provision of their health care services lags behind that of urban residents. As a result, the development of full-life-cycle services may not align with the co-occurring health care needs of both groups. Therefore, the government and society should closely monitor residents’ evolving health care needs and address any disparities. Additionally, the government should dynamically adjust health care service offerings, prioritizing network information guidance, exercise guidance, health guidance, and other essential services.

Furthermore, our results revealed a positive effect of internet use frequency on co-occurring health care needs, aligning with the findings of Li and Zhang [44]. However, their study focused on a single health care need rather than multiple health care needs. Internet platforms facilitate the rapid integration and dissemination of health-related information, allowing individuals to access a vast array of resources during their daily browsing. Additionally, access to medical websites, health apps, and WeChat public accounts enables residents to obtain previously unknown or inaccessible health information, potentially stimulating and catalyzing the emergence of multiple co-occurring health care needs [45].

At the same time, individuals with chronic diseases can use the internet for self-diagnosis, treatment planning, and prognosis management, gaining a deeper understanding of their health concerns and needs. In addition to increasing patients’ demand for diagnostic and treatment services [46], the internet facilitates social connections and communication among residents across different regions. By enabling various information-sharing mechanisms, it promotes health information dissemination and raises residents’ awareness of their health care needs [34]. Therefore, the Chinese government should continue advancing internet technology and expanding its use to deliver accurate health information through big data platforms, ultimately enhancing residents’ access to health care services and overall well-being.

Our empirical study found that physical exercise is one of the pathways through which internet use frequency influences co-occurring health care needs. This pathway suggests that higher internet use frequency increases co-occurring health care needs by promoting physical exercise among residents. This finding aligns with those of Zhang and Zhang [47] and Minto et al [48] and can be attributed to the self-selection characteristics of internet use [36], where individuals selectively use the internet based on their needs and conditions. In turn, this self-selection empowers individuals to access professional health information, manage their well-being, improve their physical exercise, and spread the benefits of online health resources. Increased physical activity is likely to drive demand for exercise guidance, as well as dietary, work, and relaxation advice, fostering the emergence of co-occurring health care needs. However, some studies argue that excessive internet use may lead to overreliance on technology and negatively impact healthy lifestyles, potentially reducing physical activity and increasing smoking and alcohol consumption [35]. Although we did not observe an inhibitory effect of internet use frequency on physical exercise, the potential negative impact of irrational internet use cannot be overlooked. Therefore, the Chinese government should enhance educational programs and public awareness campaigns on responsible internet usage, strengthen the regulation and standardization of online health information, and curate high-quality sources to mitigate potential risks. Promoting these measures can encourage healthier lifestyles, improve physical activity levels, and help residents effectively address their co-occurring health care needs.

Our study further revealed that health status mediates the relationship between internet use frequency and co-occurring health care needs. Specifically, frequent internet use appears to reduce co-occurring health care needs by improving residents’ overall health. This positive impact aligns with Zhang et al [49], who found that frequent internet use significantly enhances physical and mental well-being. Traditionally, health information has been monopolized by professionals, limiting individuals’ ability to assess their health status promptly or make necessary adjustments for improvement [50]. However, increased access to online health resources empowers individuals to take a more proactive role in managing their health. A key advantage of internet technology is its ability to bridge the gap in health information, enabling individuals to make informed decisions about their health by accessing vast online resources [51]. Frequent internet use facilitates access to valuable health information, helping residents optimize personal health care services and improve health outcomes [33]. Additionally, high internet use frequency can expand social networks, enhance social participation, and foster interactions, ultimately improving health and alleviating health-related concerns. This, in turn, may reduce reliance on traditional health care services [52].

The findings of this study offer valuable directions for future research on the relationship between internet use frequency and health care needs. Special attention should be given to the mediating role of health status and the function of internet technology as a reliable information channel that can enhance public health. Additionally, evaluating the impact of health status on health care needs is crucial for addressing co-occurring health care needs effectively and identifying variations in their prevalence based on residents’ health conditions. Unnecessary services that do not contribute to health improvement should be avoided. Instead, priority should be given to delivering essential health care services that address residents’ specific needs in this new era.

### Limitations

Our study has several limitations. First, although we incorporated household- and individual-level variables, other factors may influence co-occurring health care needs, potentially affecting the robustness of our results. Future studies should consider additional covariates to control for confounding effects, such as digital health literacy, types of chronic diseases, and preexisting conditions. Second, we measured internet use solely by frequency, without accounting for duration, content quality, or specific usage contexts. However, this measure does not fully capture the scope of internet use. As a result of data limitations, we could not include multiple variables to assess different aspects of internet use. Future studies should incorporate multidimensional measures to provide a more comprehensive understanding of its effects.

Third, because our study relied on cross-sectional data, we could not establish a causal relationship between internet use frequency and co-occurring health care needs, limiting our ability to address potential reverse causality. Future research should employ longitudinal panel data and utilize methods such as instrumental variables and fixed-effect models to better explore causality and mitigate reverse causality concerns.

Fourth, because our study utilized survey data from 2018 to examine the relationship between internet use frequency and co-occurring health care needs, our findings may not fully capture the latest trends in digital health. Given the rapid advancement and widespread adoption of internet technology, future research should incorporate more recent data to reassess this relationship. Finally, our sample was limited to 4 counties, which may affect the generalizability of our findings at the national level. Future studies should enhance regional diversity or adjust sampling weights to improve the representativeness of the results.

### Conclusions

Our study examines the association between internet use frequency and co-occurring health care needs among Chinese residents, highlighting the pathways that shape this relationship. The findings suggest that frequent internet use enhances residents’ access to health-related information, increasing their awareness of and demand for health care services. Furthermore, this relationship is mediated by 2 key factors—physical exercise and health status—forming 2 distinct pathways: (1) internet use frequency → physical exercise → co-occurring health care needs and (2) internet use frequency → health status → co-occurring health care needs.

In conclusion, our findings provide evidence of the beneficial spillover effect of online health information on individuals’ overall health. Given the ever-evolving health care needs of the general population, internet-based technologies can help adapt health care service content and delivery to meet public demands effectively. Additionally, attention should be given to weak internet infrastructure in rural areas and the digital exclusion of older adults. Therefore, the government must prioritize community digital health training, expand rural 5G coverage, and enhance digital health support for rural and older adult populations.

## Appendix

Multimedia Appendix 1 Results of the collinearity test of variables.

Multimedia Appendix 2 Histogram and normal density.

## Data Availability

The data sets generated and analyzed during this study are not publicly available due to the limited availability of data supporting the findings, but are available from the corresponding author on reasonable request.
