# Supplementary material for: The Association Between Internet Use and Co-occurring Health Care Needs: Cross-Sectional Study in China
Source: J Med Internet Res. 2025 Apr 17;27:e67484. doi: 10.2196/67484 (PMC12046273; doi:10.2196/67484)
Supplement: Multimedia Appendix 1 [file jmir_v27i1e67484_app1.docx]

**Multimedia Appendix 1. Results of collinearity test of variables**

| Variable | VIF |
| --- | --- |
| Co-occurring healthcare needs | 1.700 |
| Frequency of internet use | 1.460 |
| Gender | 1.060 |
| Age | 1.440 |
| Marital status | 1.080 |
| Education level | 1.960 |
| Household registration | 1.120 |
| Employment status | 1.220 |
| Income level | 1.090 |
| Family type | 1.150 |
| Health insurance | 1.010 |
| Service accessibility | 1.110 |
| Mean VIF | 1.280 |
