# Supplementary material for: The Association Between Internet Use and Co-occurring Health Care Needs: Cross-Sectional Study in China
Source: J Med Internet Res. 2025 Apr 17;27:e67484. doi: 10.2196/67484 (PMC12046273; doi:10.2196/67484)
Supplement: Multimedia Appendix 2 [file jmir_v27i1e67484_app2.docx]

**Multimedia Appendix 2. Histogram and normal density**
